# Relative Contributions of *Halobacteriovorax* and Bacteriophage to Bacterial Cell Death under Various Environmental Conditions

**DOI:** 10.1128/mBio.01202-18

**Published:** 2018-08-07

**Authors:** Huan Chen, Edward A. Laws, Julio L. Martin, Timkhite-Kulu Berhane, Paul A. Gulig, Henry N. Williams

**Affiliations:** a National High Magnetic Field Laboratory, Florida State University, Tallahassee, Florida, USA; b Department of Environmental Sciences, College of the Coast & Environment, Louisiana State University, Baton Rouge, Louisiana, USA; c Department of Molecular Genetics and Microbiology, College of Medicine, University of Florida, Gainesville, Florida, USA; d School of the Environment, Florida Agricultural and Mechanical University, Tallahassee, Florida, USA; University of Hawaii at Manoa

**Keywords:** *Bdellovibrio* and like organisms, *Halobacteriovorax*, bacterial mortality, bacteriophage, microbial food web, predator-prey interactions

## Abstract

The role of protists and bacteriophages in bacterial predation in the microbial food web has been well studied. There is mounting evidence that *Bdellovibrio* and like organisms (BALOs) also contribute to bacterial mortality and, in some cases, more so than bacteriophages. A full understanding of the ecologic function of the microbial food web requires recognition of all major predators and the magnitude of each predator’s contribution. Here we investigated the contribution of *Halobacteriovorax*, one of the BALOs, and bacteriophages when incubated with their common prey, Vibrio vulnificus, in a seawater microcosm. We observed that *Halobacteriovorax* was the greatest responder to the prey, increasing 18-fold with a simultaneous 4.4-log-unit reduction of V. vulnificus at 40 h, whereas the bacteriophage population showed no significant increase. In subsequent experiments to formulate a medium that would support the predatory activities and replication of both predators, low-nutrient media favored the predation and replication of the *Halobacteriovorax*, whereas higher-nutrient media enhanced phage growth. The greatest prey reduction and replication of both *Halobacteriovorax* and phage were observed in media with moderate nutrient levels. Additional experiments show that the predatory activities of both predators were influenced by environmental conditions, specifically, temperature and salinity. The two predators combined exerted greater control on V. vulnificus, a synergism that may be exploited for practical applications to reduce bacterial populations. These findings suggest that along with bacteriophage and protists, *Halobacteriovorax* has the potential to have a prominent role in bacterial mortality and cycling of nutrients, two vital ecologic functions.

## IMPORTANCE

Although much has been reported about the marine microbial food web and the role of micropredators, specifically viruses and protists, the contribution of *Bdellovibrio*-like predators has largely been ignored, posing a major gap in understanding food web processes. A complete scenario of the microbial food web cannot be developed until the roles of all major micropredators and the magnitude of their contributions to bacterial mortality, structuring of microbial communities, and cycling of nutrients are assessed. Here we show compelling evidence that *Halobacteriovorax*, a predatory bacterium, is a significant contributor to bacterial death and, in some cases, may rival viruses as agents of bacterial mortality. These results advance current understanding of the microbial loop and top-down control on the bacterial community.

## INTRODUCTION

*Halobacteriovorax* (1), a genus of the predatory bacteria, *Bdellovibrio* and like organisms (BALOs), attacks and lyses many Gram-negative bacteria and is ubiquitous in saltwater environments. Up to 85% of cultivable bacteria in estuarine systems have been reported to be susceptible to these predators (2). Although bacteriophages (phages) are considered to be major contributors to bacterial mortality and cycling of nutrients through the microbial loop, recent evidence shows a similar role in bacterial mortality for *Halobacteriovorax*, which has been largely ignored (3–5).

There are distinct differences between predation by viruses and *Halobacteriovorax*. Typically, phages are prey specific, infecting a single species or strain, and their prey bacteria can rapidly develop resistance (6). BALOs, on the other hand, typically have a relatively wide prey range (7–10). Limited evidence of predator-prey antagonistic coevolution suggests that prey evolved to be either superresistant to predation or moderately resistant, coevolving with the predator depending on the ecologic conditions (11). During the intracellular growth and replication cycle, phages do not utilize their prey’s cytoplasmic material, and most of it is released upon lysis of the prey into the ambient water as dissolved organic material (DOM) (12). In contrast, BALOs typically consume much of the prey’s cytoplasmic material, leaving little DOM to be released following prey cell lysis. Thus, BALOs sequester nutrients from bacteria that would have been released into the environment for higher trophic levels. In this way, BALOs influence nutrient cycling within the microbial loop in a much different way than phages do. Evidence strongly suggests that BALOs exert a potential sideways control on nutrient cycling (13, 14).

Virus abundance in aquatic systems is typically magnitudes (millions of virus per ml) higher than that of BALOs, which is reported to be between 10^3^ and 10^6^ cells per ml (15, 16). Phages have large burst sizes (on average, 24 but as high as 725) (17, 18), whereas the average burst size of BALOs is reported to be between 1.8 and 8.5 particles per prey cell (19–21), although numbers as high as >20 in filamentous multinucleate Escherichia coli cells have been reported (22). Second, phages can remain stable for years without the support of prey, whereas BALOs typically lose viability within several hours if the prey is not available (8, 23).

Also, phages attack rapidly growing and dominant bacterial strains in aquatic ecosystems (24, 25), whereas BALOs can efficiently prey on bacteria in the stationary growth phase (26). A recent investigation found that both predators can occur in the same bacterial cell and successfully reproduce themselves (27). This is an especially valuable mechanism when the prey is in short supply, and the survival of the predators may be at stake.

Both BALOs and phages have been examined for use as therapeutic agents for reducing Gram-negative bacterial infections in animals and humans; some successes have been reported (28–34). Previous studies have compared the contributions of phages and protists to bacterial mortality (35–38). We recently reported the responses of native phages and *Halobacteriovorax* in environmental water samples to an influx of Vibrio parahaemolyticus (3). However, we are not aware of any reports comparing the predatory behavior of a specific phage and *Halobacteriovorax* against the same prey bacterium. In this study, we compared both growth and predation rates of a specific phage and *Halobacteriovorax* strain when mixed with a common prey bacterium, Vibrio vulnificus, in laboratory microcosms. Subsequent experiments testing the relative contributions of *Halobacteriovorax* and phages to bacterial cell death at various temperature and salinity conditions were conducted using the most appropriate medium found for the growth of both agents. We expect the study design will show whether benefits to predation of bacteria accrue as an environmental service with both predators involved as opposed to only one of them. We predict the results from this study will advance understanding of bacterial predation and mortality and the role of *Halobacteriovorax* compared to phages.

## RESULTS

The objective of the initial, foundation experiment was to show the predator-prey dynamics of *Halobacteriovorax* and phage and their prey in a simulated natural seawater system. In this experiment, equal numbers of *Halobacteriovorax* and phages were inoculated into respective microcosm suspensions of the prey, Vibrio vulnificus, in seawater to establish the *Halobacteriovorax* control microcosm (*Halobacteriovorax* plus V. vulnificus [*HBx+Vv*]) and the phage control microcosm (phages+*Vv*). The test microcosm consisted of both predators and prey (*HBx+*phages*+Vv*).

**FIG 1:**
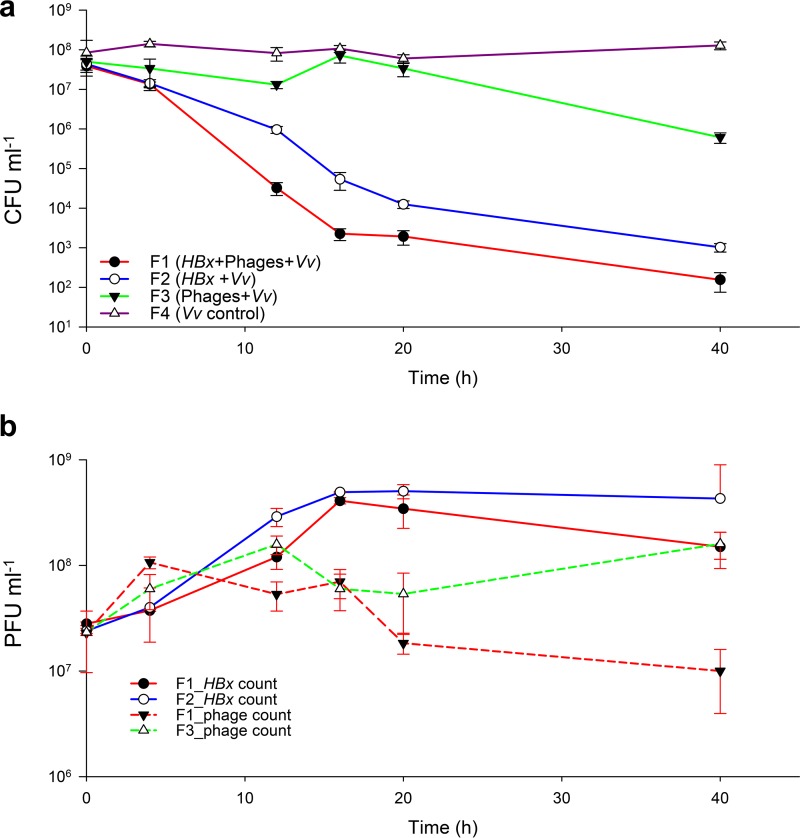
Kinetics of the lysis of prey cells (a) and growth dynamics of *Halobacteriovorax* and phage on V. vulnificus prey (b) over a 40-h period in test (with *Halobacteriovorax* plus phages plus V. vulnificus [*HBx+*phages+*Vv*]) and control (with either predator or no predators) microcosms. F1 (*HBx+*phages+*Vv*) designates the microcosm with both *Halobacteriovorax* and phage predators. F2 and F3 are the microcosms consisting of V. vulnificus and either *Halobacteriovorax* or phages, respectively. F4 is the microcosm with prey V. vulnificus only. Predator and prey counts were obtained in triplicate. Error bars are standard errors from three independent experiments.

The results from the test microcosm showed that both *Halobacteriovorax* and phages reduced the abundance of the prey significantly (*P* < 0.01 by *t* test) over 0 to 40 h. However, at 40 h, prey reduction was much greater by the *Halobacteriovorax* (*P* < 0.001 by *t* test) than by the phage (Fig. 1).

Correlated to its rapid reduction of prey, *Halobacteriovorax* responded the most to V. vulnificus in the *HBx*+phages*+Vv* test microcosm, increasing 10-fold in PFUs, which then gradually declined, reflecting a decline in growth rate after an incubation of 16 h, likely due to the low number of prey remaining to support its growth (Fig. 1b). The fact that a similar response was observed in the *HBx+Vv* control microcosm suggests that V. vulnificus predation in the test microcosm was due largely to *Halobacteriovorax*. In contrast, there was no net increase of phage at 40 h relative to the number at 0 h. Only a fivefold increase in phage was observed in the phages+*Vv* control microcosm. The calculated rates of change of the predators and prey in the respective microcosms are shown in Table 1. The difference between the predator growth rate in the *HBx+Vv* and *HBx+*Phage+*Vv* microcosms was not significantly different (*P* > 0.05), which suggests that if there were any interactions between *Halobacteriovorax* and phages in the latter microcosm, they did not affect the results.

**TABLE 1:** Calculated rates of change of the predators *Halobacteriovorax* and phage and prey V. vulnificus in the respective microcosms^a^

Microcosm	Predator growth rate (h^−1^) (*P* value)	Prey reduction rate (h^−1^) (*P* value)
HBx+Vv	0.2 ± 0.07 (*P* = 0.007)	−0.4 ± 0.24 (*P* = 0.017)
Phage+Vv	Not significant	−0.1 ± 0.098 (*P* = 0.047)
HBx+Phage+Vv	0.16 ± 0.12 for *HBx* (*P* = 0.026), not significant for phage	−0.64 ± 0.26 (*P* = 0.009)

a HBx, *Halobacteriovorax*; Vv, *V*. *vulnificus*.

The results of the predator-prey modeling exercise (see Fig. S1 in the supplemental material) produced statistically significant (*P* < 0.05) predation rates (the difference between the V. vulnificus growth rates in the control microcosm without predators and the V. vulnificus net growth rates in the presence of predators in the test microcosm) only in the case of the *HBx+Vv* control microcosm. In that case, the *Halobacteriovorax* predation rate was 8.4 × 10^−10^ ml h^−1^ *HBx*^−1^; *Vo*, the threshold prey concentration below which predation ceased, was 1.4 × 10^4^ V. vulnificus ml^−1^; and the natural V. vulnificus mortality rate (*Mv*) was 0.26 h^−1^.

**FIG 2:**
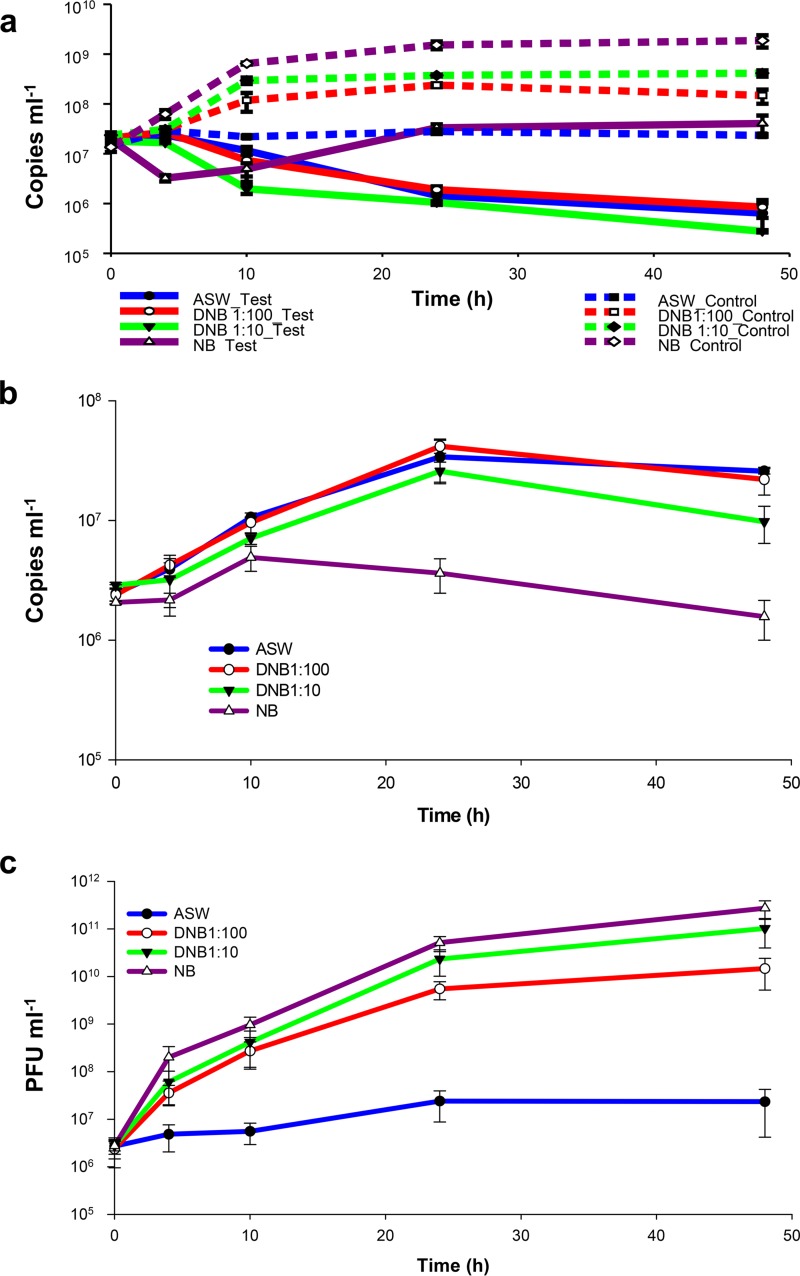
Effects of nutrients on predation on V. vulnificus by *Halobacteriovorax* and phage in combination. (a) Time course changes of V. vulnificus abundance in the test (with both predators [solid lines]) and control microcosms (prey only [broken lines]) with different nutrient concentrations as measured by qPCR assays. (b and C) Growth kinetics of *Halobacteriovorax* (b) and phages (c) on V. vulnificus in media with different nutrient concentrations. Values are means of triplicate samples. Error bars represent the standard deviations of the means (*n* = 3).

10.1128/mBio.01202-18.1 FIG S1  Results of the predator-prey model in the *Vv+HBx* microcosm. (a) Logarithms of the Vibrio vulnificus concentrations. The interpolated values were used to calculate the rate of change of the logarithms. (b) Rates of change estimated from panel a (measured) with the values calculated from a multiple linear regression model (equation 2). Because the V. vulnificus concentration at 40 h was significantly (*P* < 0.05) lower than the threshold V. vulnificus concentration, only the data from 0 to 20 h were included in the multiple linear regression analysis. The lack of statistical significance in the cases of the other two microcosms reflects scatter in the data and, in particular, the small number of degrees of freedom associated with the multiple linear regression model. In the case of the *Vv+HBx* microcosm, there were only five time points included in the analysis, and the model contained three coefficients. Hence, there were only two degrees of freedom and the fact that the model parameters were all statistically significant reflects the excellent fit to the data (Fig. 2b). Download FIG S1, TIF file, 0.3 MB. Copyright © 2018 Chen et al. 2018 Chen et al. This content is distributed under the terms of the Creative Commons Attribution 4.0 International license.

Note that the net rate of change of V. vulnificus was more negative when both *Halobacteriovorax* and phage were present than when only *Halobacteriovorax* was present, but the difference was marginally significant at the 95% confidence level. These growth rates came from analysis of the first 20 h of the experiments (*Vv* > *Vo*), except for the *Vv*+phage experiment, which was calculated for all 40 h.

The results of the foundation experiment described above show that viral growth was weak relative to *Halobacteriovorax* growth. This could be due to the possibility that optimal phage replication requires actively growing prey, which was not the case with the V. vulnificus in the artificial seawater (ASW) medium used. To test this scenario and to find a culture medium formulation that would promote optimal growth of both phage and *Halobacteriovorax* for future studies, *Halobacteriovorax*, phage, and prey combinations were grown in ASW medium supplemented with a range of nutrient concentrations. The results show that nutrient concentrations did impact predation and growth of the predators and prey. Irrespective of the nutrient concentration in the medium, the predation activity by phage and *Halobacteriovorax* on V. vulnificus led to significant decreases in the V. vulnificus population (*P* < 0.01 by *t* test) compared to the control microcosm without predators (Fig. 2). In the high-nutrient medium, nutrient broth (NB), V. vulnificus increased 138-fold in the control microcosm with no predators, whereas in the test microcosm with predators, only a twofold increase was observed between 0 and 48 h. In the low-to-moderate nutrient concentration media, including ASW (70% artificial sea water), DNB 1:100 (full-strength NB diluted 100 times), and DNB 1:10 (full-strength NB diluted 10 times), the predators reduced the population of V. vulnificus (*P* < 0.05 by *t *test). Because the maximum reduction (3.17 log units) occurred in the microcosm with DNB 1:10 at 48 h (Fig. 2a), this formulation was considered optimal.

Both *Halobacteriovorax* and phage grew somewhat similarly on V. vulnificus in DNB 1:10 and DNB 1:100 media (*P* > 0.05 by *t* test). The greatest differences between the growth of the two predators were observed in ASW, in which the *Halobacteriovorax* grew at its maximum and phage at its minimum, and in NB, in which the phage increase was at its maximum and the *Halobacteriovorax* at its minimum (Fig. 2b and c and see Fig. S2 in the supplemental material).

10.1128/mBio.01202-18.2 FIG S2  Growth and predation rates as a function of nutrient enrichment. The growth rates in panels A, C, and D were equated to the slopes of straight lines fit to the logarithms of the numbers of Vibrio vulnificus (*Vv*), *Halobacteriovorax* (*HBx*), and phage, respectively, versus time in the microcosms during the 40-h duration of the experiments. The predation rates in panel B are the differences between the growth rates in panel A and the net rates of change of the V. vulnificus in the predator-prey experiments. The latter were equated to the slopes of straight lines fit to the logarithms of the numbers of V. vulnificus versus time in the predator-prey experiments. Download FIG S2, TIF file, 0.05 MB. Copyright © 2018 Chen et al. 2018 Chen et al. This content is distributed under the terms of the Creative Commons Attribution 4.0 International license.

The growth of *Halobacteriovorax* was similar in the three media with low-to-moderate nutrient levels, ASW, DNB 1:100, and DNB 1:10 (not significantly different; *P* > 0.05 by analysis of variance [ANOVA]) (Fig. 2b and Fig. S2). *Halobacteriovorax* did not show a significant increase in full-strength nutrient broth (*P* > 0.05 by ANOVA) (Fig. 2b and Fig. S2).

Although phages did not replicate well on V. vulnificus in ASW, consistent with the results of the previous experiment, they were highly active in the other three media with higher nutrient concentrations, reaching 10^10^ to 10^11^ PFU ml^−1^ (Fig. 2c and Fig. S2). These higher-nutrient media promoted increased growth of the prey bacterium, which favored higher production of phage (Fig. 2c).

Not surprisingly, V. vulnificus growth rates in the control microcosms were positively correlated with nutrient enrichment and were virtually zero in ASW (Fig. S2a). The difference between the V. vulnificus control growth rates and the V. vulnificus net growth rates in the presence of predators was equal to the predation rate on V. vulnificus (Fig. S2b). The fact that the *Halobacteriovorax* and V. vulnificus growth rates in Fig. S2b and c are negatively correlated with each other are consistent with *Halobacteriovorax* predation on V. vulnificus. In contrast, the growth rates of V. vulnificus and phage in Fig. S2b and d were positively correlated with each other, which is inconsistent with the phage being a major predator on V. vulnificus. The implication is that *Halobacteriovorax* accounted for most of the predation on V. vulnificus in these experiments.

**FIG 3:**
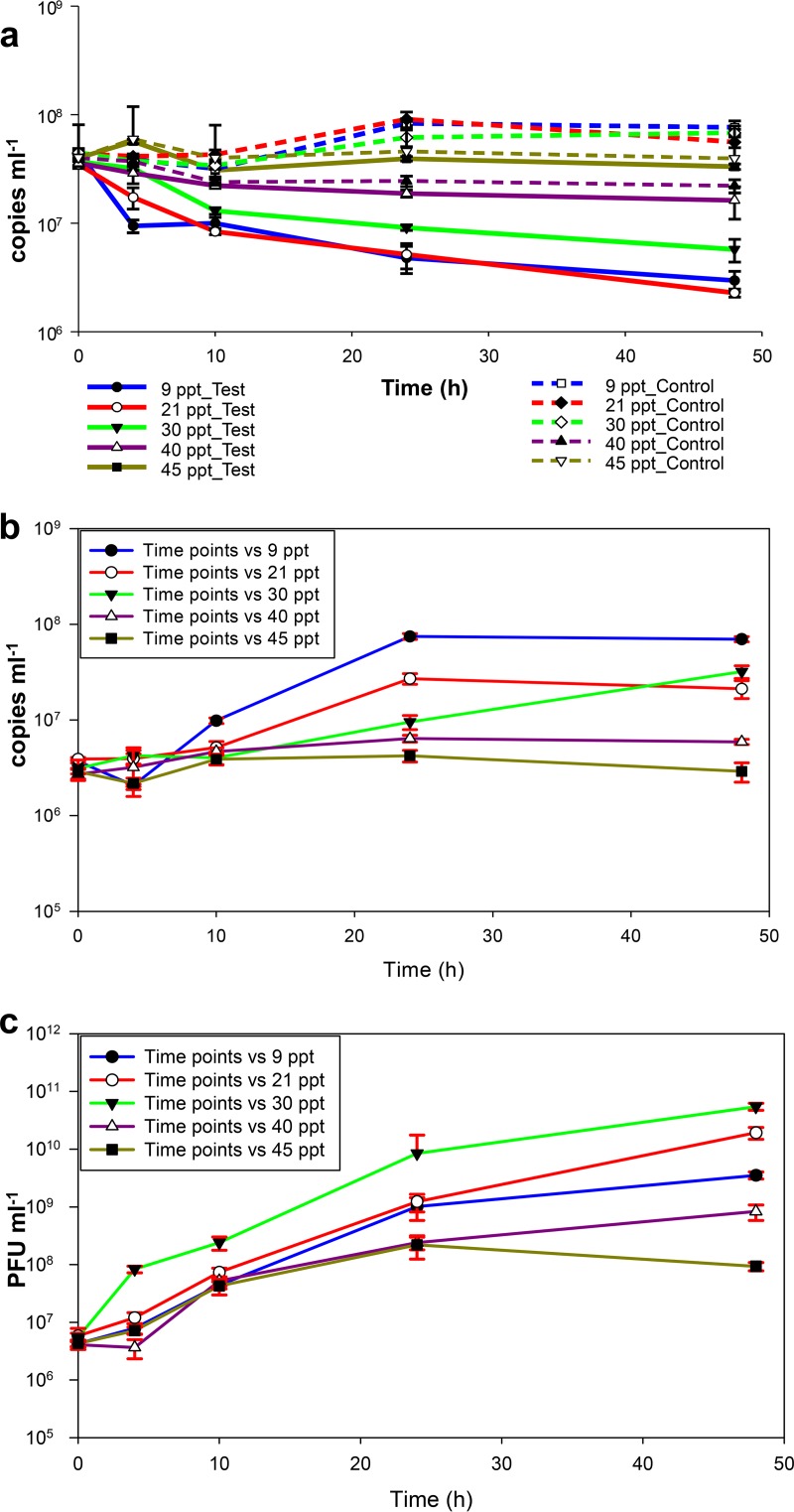
Effects of salt concentrations on predation on V. vulnificus by *Halobacteriovorax* and phage in combination in DNB 1:10. (a) Time course changes in V. vulnificus abundance in the test (both predators [solid lines]) and control microcosms (prey only [broken lines]) for various salt concentrations as measured by qPCR assays. The abundance of V. vulnificus in the control microcosms remained stable (not significantly different [*P* > 0.05 by ANOVA]). (b and c) Growth kinetics of *Halobacteriovorax* (b) and phage (c) on V. vulnificus at different salt concentrations. Values are means for triplicate samples. Error bars represent the standard deviations of the mean (*n* = 3).

Having found a suitable medium for predation and replication of both predators, we proceeded to test the effects of salt concentration and temperature on predation. The results of different salt concentrations on predation on V. vulnificus by *Halobacteriovorax* and phages and growth of the predators are summarized in Fig. 3 and Fig. S3 in the supplemental material. V. vulnificus growth rates in the control microcosm were negatively correlated with salt concentrations (Fig. S3a) and were negative at salt concentrations of 40 and 45 ppt. The predation rates on V. vulnificus were negatively correlated with salt concentration (Fig. S3b) and were close to zero at salt concentrations of 40 and 45 ppt, the indication being that predation was virtually nil at the two highest salt concentrations. The highest *Halobacteriovorax* growth rate (Fig. S3c) was associated with the highest predation rate on V. vulnificus (Fig. S3b), and the two lowest *Halobacteriovorax* growth rates, which were close to zero, were associated with predation rates on V. vulnificus that were also close to zero. Both phage growth rates (Fig. S3d) and the predation rates on V. vulnificus were negatively correlated with salt concentrations from 30 to 45 ppt. However, the correlation was positive for salt concentrations between 9 and 30 ppt. The implication is that the relative contribution of phage predation to total predation was greater at the two highest salt concentrations.

10.1128/mBio.01202-18.3 FIG S3  Growth rates of Vibrio vulnificus (*Vv*), *Halobacteriovorax* (*HBx*), and phage and predation rates on V. vulnificus as a function of the salt concentration in DNB 1:10 medium (full-strength NB diluted 10 times) with various salt concentrations. Rates were calculated in the same way as in Fig. 2. The straight line in panel c is a linear regression fit to the data. Download FIG S3, TIF file, 0.1 MB. Copyright © 2018 Chen et al. 2018 Chen et al. This content is distributed under the terms of the Creative Commons Attribution 4.0 International license.

**FIG 4:**
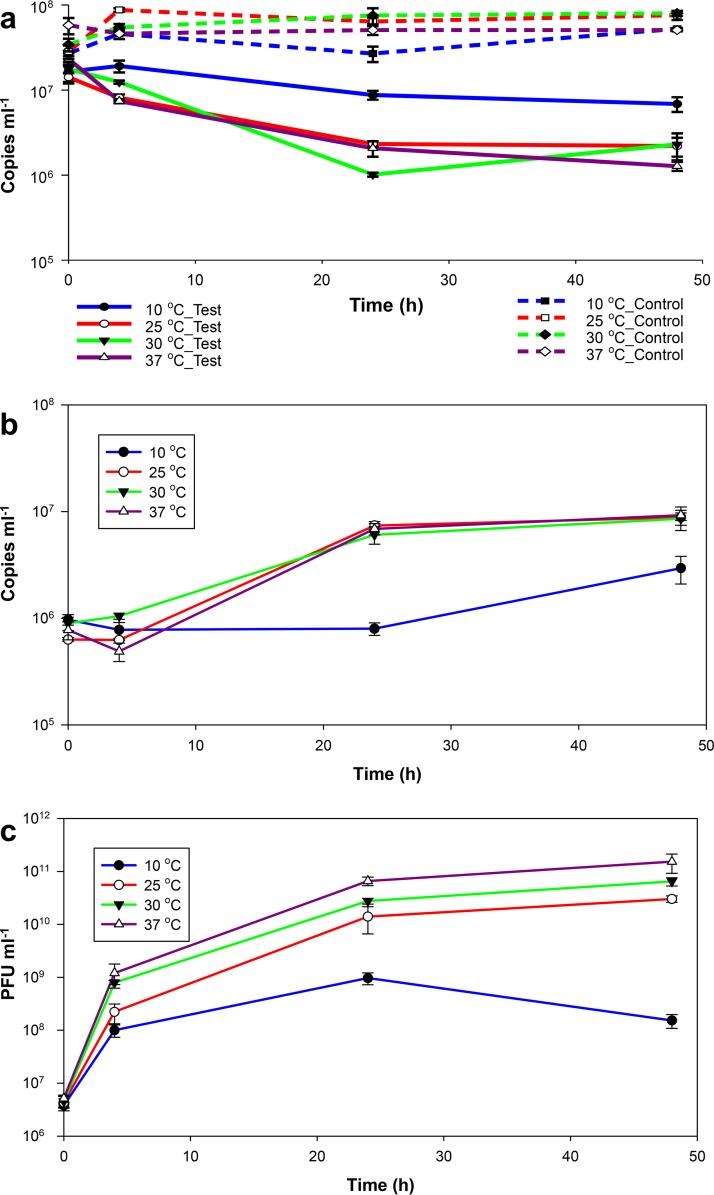
Effect of temperature on predation on V. vulnificus by *Halobacteriovorax* and phage in combination in DNB 1:10. (a) Time course changes in V. vulnificus abundance in the test (both predators [solid lines]) and control microcosms (prey only [broken lines]) at different temperatures as measured by qPCR assays. (b and c) Growth kinetics of *Halobacteriovorax* (b) and phage (c) on V. vulnificus at different temperatures. Values are means for triplicate samples. Error bars represent the standard deviations of the means (*n* = 3).

Temperature also showed an effect on predation. The reductions of V. vulnificus by predators at 48 h were significant (*P* < 0.05 by ANOVA; *P* < 0.05 by Holm-Sidak test) for all temperatures tested, 10°C, 25°C, 30°C, and 37°C. The levels of prey reduction were similar at 25°C, 30°C, and 37°C but substantially lower at 10°C (Fig. 4). V. vulnificus abundance in the control microcosms remained stable at all temperatures (not significantly different [*P* > 0.05 by ANOVA]).

The growth of *Halobacteriovorax* at 25°C, 30°C, and 37°C was not significantly different (*P* > 0.05 by ANOVA). Phage production was higher as temperature increased. The growth of both *Halobacteriovorax* and phage was substantially slower at 10°C. The abundance of *Halobacteriovorax* remained stable in the first 24 h at 10°C and then increased slightly (0.6 log unit) after 48 h of incubation (Fig. 4a). Conversely, the phage population increased during the first 24 h and then decreased (Fig. 4b).

V. vulnificus control growth rates increased with temperature up to 30°C and declined abruptly at 37°C (see Fig. S4a in the supplemental material). Predation rates on V. vulnificus were positively correlated with temperature (Fig. S4b), as were the growth rates of *Halobacteriovorax* (Fig. S4c) and phage (Fig. S4d). The implication is that the impact of predation on V. vulnificus was positively correlated with temperature. This has been shown by Richards et al. (39) at some sites but not others. *Halobacteriovorax* was recovered over a temperature range from 5°C to 30°C. It is impossible to say from the results in Fig. 4 whether *Halobacteriovorax* or phage was a more important predator.

10.1128/mBio.01202-18.4 FIG S4  Growth rates and predation rates versus temperature in DNB1:10 medium. Rates were calculated in the same way as in Fig. 2. The straight lines in panels B to D are linear regressions fit to the data. Download FIG S4, TIF file, 0.1 MB. Copyright © 2018 Chen et al. 2018 Chen et al. This content is distributed under the terms of the Creative Commons Attribution 4.0 International license.

## DISCUSSION

Bacterial predators have been recognized and acknowledged to have critical roles in nature in controlling and shaping the structure of bacterial communities, and global biogeochemical cycling. Yet, other than bacterial viruses and protist grazers, little is known about predators of bacteria and, in particular, bacteria that prey on other bacteria. More than a dozen predatory bacteria have been reported in the literature, and perhaps many more exist (40–42). These predatory bacteria may also be important in regulating and structuring bacterial communities and nutrient cycling but have not received the attention they deserve. The most-studied predatory bacteria are the *Bdellovibrio* and like organisms (BALOs), a group consisting of several different families, genera, and phylogenetic clusters that vary in physical and physiological characteristics, prey range, and habitat.

The literature on bacterial predation, bacterial mortality, and the microbial loop has historically ignored any contribution by *Halobacteriovorax* and other BALOs. The findings reported here show *Halobacteriovorax* to be active predators with the potential for regulating and structuring bacterial communities and nutrient cycling in the environment. This is supported by results from other studies (4, 13, 43–46).

As reported in previous studies (3, 5), *Halobacteriovorax* has been shown in the current study to be the major factor in the mortality of V. vulnificus and V. parahaemolyticus, respectively, in seawater compared to phages. Both *Halobacteriovorax* and phages were also shown to be influenced by environmental factors and sometimes in different ways, which can be beneficial to the environment in expanding control of bacterial populations over a greater range of physical and chemical conditions (47). For example, at a low temperature (10°C), phage numbers increased during the first 24 h followed by a decline, whereas the *Halobacteriovorax* numbers did not begin to increase until after 24 h. This “synchronization” illustrates how the combined growth of the phage and *Halobacteriovorax* resulted in a continuous decline in prey population over a 48-h period, which neither would have accomplished alone. A similar effect was observed with the effect of salt concentration.

For optimal growth of *Halobacteriovorax* and other BALOs, a low-nutrient medium is typically required. Phages, on the other hand, are typically grown in enriched media that supports the high metabolic activity of their prey. For any comparative growth studies on *Halobacteriovorax* and phage, a suitable medium for optimal growth of both agents is required. We observed in this study that a 1:10 dilute formulation of nutrient broth supported optimal growth of both *Halobacteriovorax* and phage. This seemingly simple development is a significant advance in the capability to conduct comparative studies between these two important predators.

The response of the *Halobacteriovorax* and phage to different nutrient concentrations in growth media suggest an advantage for *Halobacteriovorax* in natural bodies of waters (typically of low nutrients) as reported in our previous work (3). Optimal conditions for BALO and phage predation and replication intersect with the conditions under which the V. vulnificus prey grow in a predictable manner that can be modeled *in silico*.

In this study, experimental microcosms included both *Halobacteriovorax* and phages with a common prey. Under such conditions, there is the possibility for interactions among the predators to either interfere with or enhance predation. We observed no direct evidence that such interactions occurred, although when both predators are present, the decline of the prey may be slightly greater. It is also possible that in the high-nutrient environments tested, the phages may rapidly decimate the prey population, leaving fewer numbers of cells for the *Halobacteriovorax* to prey upon. In the low-nutrient media, which favors rapid growth of *Halobacteriovorax*, the predators may lyse the prey, leaving insufficient prey to support phage infection and replication. The interactions of predators and the consequential effects on predation dynamics require further scrutiny.

Our findings challenge the current paradigm that positions phages and protists as the major predators of bacteria by providing strong evidence showing the potential for BALOs to also have a prominent role in bacterial mortality, and by implication, the cycling of nutrients through the microbial loop, two important ecologic services. *Halobacteriovorax* adds a new dimension to nutrient cycling. Unlike phages, which lyse their prey, causing the release of prey cellular contents into the environment (48, 49), *Halobacteriovorax* and other BALOs consume much of the prey cellular content prior to lysis (50), thereby preserving prey nutrients and avoiding their immediate loss into the extracellular environment.

The results of this investigation establish a platform for future studies involving multiple predators, including the BALOs, to further advance research and knowledge on bacterial mortality and nutrient cycling. Such an approach has been suggested by Johnke et al. (47). The collective observations made in this study advance our understanding of the different roles of various predators in bacterial mortality and the impact of the environment on their predatory functions.

## MATERIALS AND METHODS

### Bacterial and phage strains and culture conditions.

Vibrio vulnificus FLA042 (27) was selected as prey for the bacterial and viral predators, *Halobacteriovorax* cluster IX and bacteriophage CK2, respectively. Both predators were found in previous studies to be relatively efficient in predation on V. vulnificus compared to other predators tested (44, 51, 52). A second prey, *V. vulnificus* strain MO6-24/O (53), was used as prey specifically to quantify mortality caused by *Halobacteriovorax* because *V. vulnificus* strain MO6-24/O is resistant to the CK2 phage, which does not form plaques on it. Plaques observed on lawns of strain MO6-24/O, therefore, resulted exclusively from *Halobacteriovorax* lysis. Laboratory tests confirmed that phage CK2 could form clear plaques on V. vulnificus FLA042 but not on strain MO6-24/O, whereas BALOs could form plaques on both V. vulnificus strains at the same rate (*P* > 0.05 by *t* test) using the Pp20 double agar overlay technique.

Suspensions of the prey were prepared by adding 5 ml of 70% artificial seawater (ASW) (Instant Ocean; Aquarium Systems, Inc., Mentor, OH) (pH 8; salt concentration, 21 ppt) to culture plates (Difco) of an overnight culture of V. vulnificus grown on Luria-Bertani (LB) agar and suspending the colonies in the liquid. The resulting prey suspensions were transferred into sterile tubes for subsequent inoculation into flasks of artificial seawater to establish microcosms for comparing predation rates between phages and *Halobacteriovorax*. The number of prey bacteria in the prey suspension was determined by spread plating 0.1 ml of serial 10-fold diluted samples onto LB agar plates in triplicate. These plates were incubated at 37°C for 2 days, and CFUs were counted and recorded.

Active *Halobacteriovorax* cultures were grown in ASW-V. vulnificus broth and transferred weekly. To obtain a pure suspension of *Halobacteriovorax* for the predation experiments, the culture lysates of 24- to 48-h cultures were filtered consecutively through 0.45- and 0.22-µm syringe filters to remove any remaining prey. One liter of the filtrate containing the *Halobacteriovorax* cells (as determined by fluorescence microscopy) was centrifuged at 27,485 × *g* for 30 min. The pellet of predator cells was then resuspended in 6 ml of ASW. To test whether the concentrated *Halobacteriovorax* suspension was free of prey cell contamination, 0.1-ml aliquots were spread plated onto LB agar and incubated at 37°C for 2 days. The number of *Halobacteriovorax* in the suspension was determined by both plate counts and 4′,6-diamidino-2-phenylindole (DAPI) direct cell counts. Counts by the two methods were not significantly different (*P* > 0.05 by *t* test). Microscopic counts were used to enumerate and establish the desired ratio of predator and prey cells at the beginning of the predation experiments. The plate counts were recorded to assess the number of infective *Halobacteriovorax* and phage in the samples.

*Halobacteriovorax* plate counts were determined using the double agar overlay method described by Williams and Falker (54) and other investigators (10). *Halobacteriovorax* plaques on the plates were monitored and counted daily for a week.

Purified phage CK2 was maintained at 4°C. Active phage cultures were prepared, and the titer was determined by the method of Martin (51) one day before the start of the experiment. Briefly, a culture of V. vulnificus was infected with serially diluted phage and incubated for 10 min at room temperature. The resulting mixture was added with four milliliters of LB-SW soft agar, vortexed, then poured over an LB-SW plate, and the plate was incubated overnight at 37°C. The next day, the plaques were counted, and the titer was calculated. All plaques observed were a result of phage lysis because *Halobacteriovorax* could not form plaques on LB, an enriched medium, within the incubation period.

### Seawater microcosm experiments: individual and combined effects of predators on prey bacteria.

Microcosms were established to monitor the population dynamics between predator and prey and their respective abundances at selected time intervals. For the initial investigation, equal numbers of active *Halobacteriovorax* and phages in respective suspensions were inoculated into the test microcosm containing V. vulnificus suspended in 200 ml of sterilized natural seawater yielding a predator/prey ratio of 1:1:1 (F1 microcosm; *Halobacteriovorax* plus phages plus V. vulnificus [HBx+Phages+*Vv*] microcosm). Three control microcosms were established: one for monitoring the growth in dual cultures of V. vulnificus and *Halobacteriovorax* (F2; *HBx*+*Vv*), a second for monitoring the growth in dual cultures of V. vulnificus and phages (F3: phages+*Vv*), and a third for monitoring the growth of V. vulnificus prey only, without the predator amendments (F4; V. vulnificus control [*Vv* control]). Microcosms were incubated while shaking at 25°C for 40 h. Test and control microcosms were monitored by measurements of optical density (OD) values every 4 h. Aliquots of samples were removed at 0, 12, 20, and 40 h to obtain viable counts of *Halobacteriovorax*, phages, and V. vulnificus by the plating methods described above. This experiment was repeated three times.

### Predator-prey model.

In the case of the F2 microcosm, or *HBx+Vv* microcosm, we assumed that the dynamics of the V. vulnificus were described by the equation

(1) $$\frac{dVv}{dt}=-G\cdot HBx(Vv-Vo)-Mv\cdot Vv$$

where *G* is the predation rate (in milliliters per hour per *HBx*), *Vo* is the concentration of V. vulnificus below which predation by *Halobacteriovorax* ceases (i.e., *G* = 0), and *Mv* is the mortality rate (per hour) of V. vulnificus due to factors other than predation by *Halobacteriovorax*. We assumed a threshold predation concentration based on the work of Fenton et al. (20). Equation 1 may be rewritten in the form

(2) $$\frac{1}{Vv}\frac{dVv}{dt}=-Mv-G\cdot HBx+G\cdot Vo\frac{HBx}{Vv}$$

The left-hand side of equation 2 is the rate of change of the logarithm of V. vulnificus (*Vv*). The right-hand side consists of three terms, a constant term (−*Mv*), a term proportional to the concentration of *Halobacteriovorax* (−*G*), and a term proportional to the ratio of *Halobacteriovorax* to V. vulnificus (*G ⋅ Vo*). To estimate the rate of change of the logarithm, we interpolated the logarithms of the V. vulnificus concentrations at 1-h intervals with a shape-preserving piecewise cubic interpolation using MatLab software. The rates of change of the logarithms were then estimated using one-sided finite difference equations at times 0 and 40 h and time-centered finite differences at 4, 12, 16, and 20 h. The parameters −*Mv*, −*G*, and *G ⋅ Vo* were then estimated by multiple linear regression analysis of the rate of change of the logarithm of V. vulnificus with a constant term and *Halobacteriovorax* and *Halobacteriovorax*/V. vulnificus as the independent variables. The data included in the analysis consisted of data at time points where the observed V. vulnificus concentration was significantly (*P* < 0.05) greater than the calculated *Vo*. A similar approach was used in the case of the *Vv*+phage microcosm, but in that case, we assumed that there was no threshold V. vulnificus concentration below which predation ceased. The two equations were combined in the case of the *Vv+HBx*+phage microcosm:

(3) $$\frac{1}{Vv}\frac{dVv}{dt}=-Mv-G\cdot HBx+G\cdot Vo\frac{HBx}{Vv}-G'\cdot \text{phage}$$

On the basis of the results showing low predation and growth rates of bacteriophage compared to the BALOs, we considered that this could be due to the low-nutrient medium used (ASW plus prey) in the experiments. Although the ASW may be comparable to the natural environmental water, it did not support an actively growing prey bacterial population suggested as being necessary for optimal bacteriophage replication (25). This being a possibility, we sought a medium that would support the growth of both bacteriophage and *Halobacteriovorax*. Such a medium, although perhaps not representative of natural waters, would be an important advance in the capability to conduct experimental comparative studies on these two predators, including the testing of their responses to various parameters. We were especially interested in examining the *Halobacteriovorax* and phage predation activity under different environmental conditions.

### Testing growth media to support *Halobacteriovorax* and bacteriophage.

ASW, supplemented with different concentrations of nutrient, was tested for growth of *Halobacteriovorax* and phage. The nutrient concentrations included full-strength nutrient broth (NB) (Difco) and diluted nutrient broth (DNB) preparations (full-strength nutrient broth diluted 10 times [DNB 1:10] and diluted 100 times [DNB 1:100]) in ASW, and ASW with no added nutrients. For all medium preparations, the salt concentration and pH were held constant at 21 ppt and pH 8, respectively. A test microcosm of each of the four nutrient medium formulations was established. Harvested V. vulnificus culture suspensions were inoculated into each microcosm flask to an OD of 0.3, corresponding to ca. 2 × 10^7^ CFU ml^−1^. Active *Halobacteriovorax* and phage cultures were inoculated into the four test microcosms simultaneously to yield a final concentration of ca. 2 × 10^6^ PFU ml^−1^. Four additional microcosms of the respective medium formulations containing only prey served as controls. Microcosms were incubated at 25°C while shaking (130 rpm). At intervals of 0, 4, 10, 24, and 48 h, aliquots were aseptically removed from each microcosm for OD measurements. *Halobacteriovorax* and V. vulnificus in the respective microcosms were enumerated by quantitative real-time PCR (qPCR) (55) using the Bio-Rad CFX96 real-time PCR detection system (Bio-Rad, Hercules, CA). To quantify V. vulnificus, the oligonucleotide primers *Vv*hA 1973 rev (rev stands for reverse) (5′-TCG ACT GTG AGC GTT TTG TC-3′) and *Vv*hA1795 [5′-TGC CT(AG) GAT GTT TAT GGT GAG AAC-3′] were used to target the V. vulnificus cytolysin/hemolysin gene (56). Each sample was measured in triplicate. Negative extraction controls and negative controls (no template) were included. A 10-fold dilution series of a plasmid containing a fragment of the *Halobacteriovorax* 16S rRNA gene or the V. vulnificus hemolysin/cytolysin gene was used in the qPCR assay to construct a standard curve (correlation coefficient of >0.99). Phage numbers were determined by the plating method described above.

### Predation under various environmental conditions.

Experiments testing the relative contributions of *Halobacteriovorax* and phages to bacterial cell death at various temperature and salinity conditions were conducted. DNB 1:10 was selected as the culture medium for these experiments as it was found to support the optimum growth of both *Halobacteriovorax* and phage when tested against other medium formulations. We also wanted to assess whether the range of predation was expanded under various environmental conditions by the presence of the two predators versus only one predator. As in the previous experiments, qPCR was used to quantify *Halobacteriovorax* and prey in the mixed culture. The virus was quantitated by direct plating as described above. Sampling time intervals of the latter experiments were also adjusted based on the results of the experiment with the seawater microcosm.

### (i) Salt concentrations.

To measure the effects of various salt concentrations on predation by phages and *Halobacteriovorax* on the population of V. vulnificus, we used a basal medium of DNB 1:10 supplemented with 3 mM CaCl_2_ and 2 mM MgCl_2_ (57) with different NaCl salt concentrations in each batch (100 ml each). The salt concentrations were 9, 21, 30, 40, and 45 ppt, adjusted using synthetic sea salt (Instant Ocean; Aquarium Systems, Inc., Mentor, OH). The pH was adjusted to pH 8 for all microcosms. Respective suspensions of *Halobacteriovorax*, phage, and V. vulnificus were inoculated into each microcosm to yield a predator-prey ratio of 1:10. The microcosms were incubated, and aliquots were removed for quantification of bacteria and phage as described above.

### (ii) Temperature.

The impact of temperature on phage and *Halobacteriovorax* predation was examined in DNB 1:10 medium, at pH 8, a salt concentration of 21 ppt, and a predator/prey ratio of 0.1. Each set of test and control microcosms (100 ml each) was incubated on shakers (130 rpm) at temperatures set at 10°C, 15°C, 25°C, 30°C, and 37°C. Aliquots of the samples from each microcosm were removed aseptically at 0, 4, 24, and 48 h for OD measurements. Bacterial and phage counts were measured as described above and recorded.

### Statistical analysis.

Analysis of variance (ANOVA) was used followed by Holm-Sidak test to detect significant differences among the numbers (log transformed) of microbes in the various microcosm treatments. A *t* test was used to compare two groups of treatments when normality and equal variance tests were passed. These statistical analyses were performed using the Sigmastat, version 3.5, software package. The growth rates (per hour) of the V. vulnificus, *Halobacteriovorax*, and phage as functions of temperature and concentrations of nutrients and salt were estimated from the slopes of straight lines fit to the logarithms of the organism concentrations versus time. Reported error bounds are 95% confidence intervals assuming a normal distribution of errors.
