# Supplementary figures and images for: Relative Contributions of Halobacteriovorax and Bacteriophage to Bacterial Cell Death under Various Environmental Conditions
Source: mBio. 2018 Aug 7;9(4):e01202-18. doi: 10.1128/mBio.01202-18 (PMC6083911; doi:10.1128/mBio.01202-18)

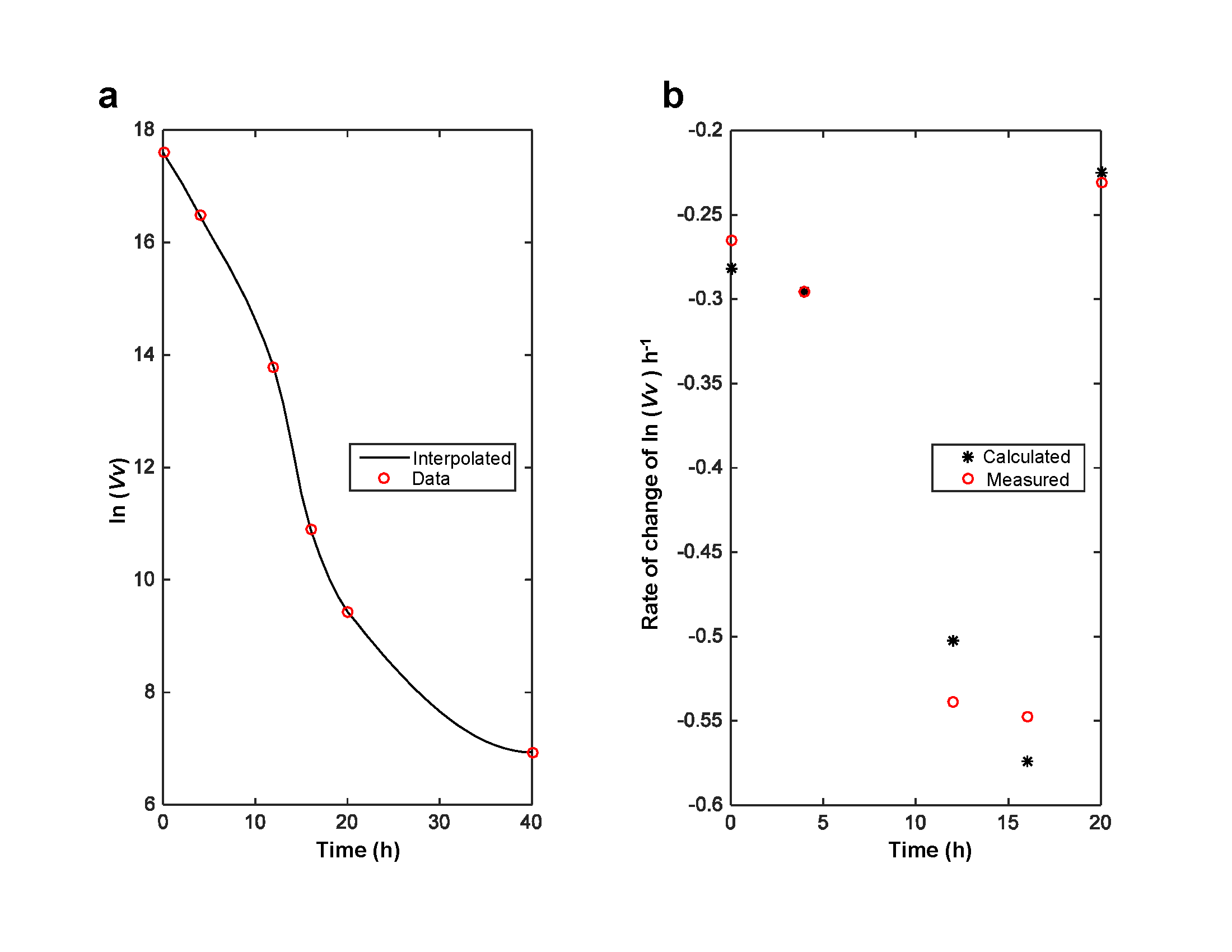

Supplement: FIG S1 [file mbo004184005sf1.tif]

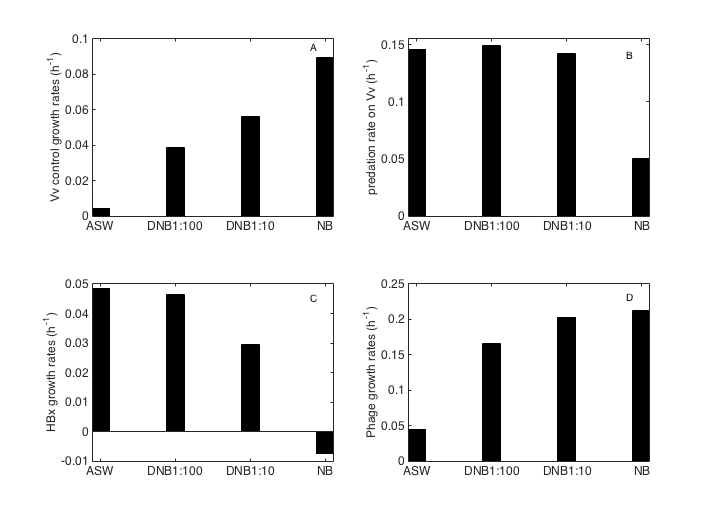

Supplement: FIG S2 [file mbo004184005sf2.tif]

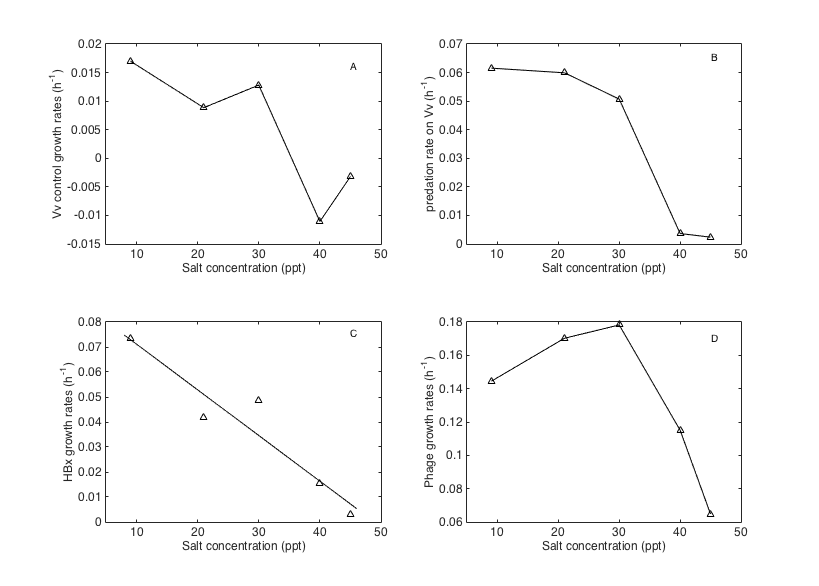

Supplement: FIG S3 [file mbo004184005sf3.tif]

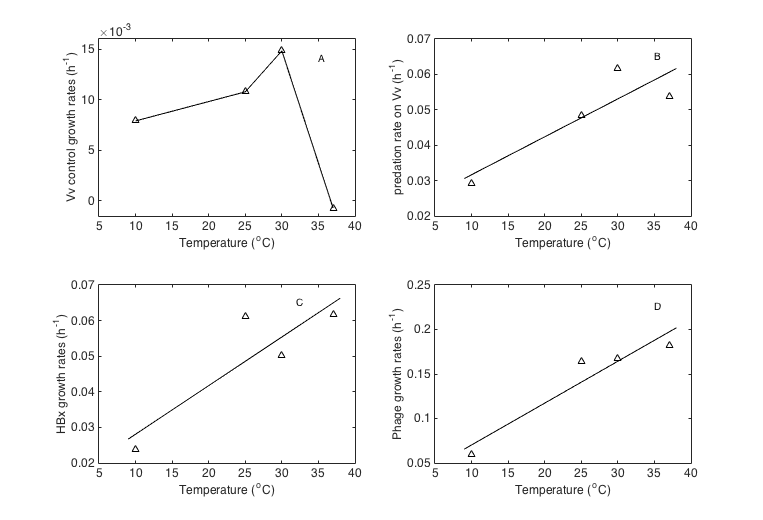

Supplement: FIG S4 [file mbo004184005sf4.tif]
